# Clinical and Pathological Characteristics of Patients With Nonproteinuric Diabetic Nephropathy

**DOI:** 10.3389/fendo.2021.761386

**Published:** 2021-10-26

**Authors:** Dong-Yuan Chang, Meng-Rui Li, Xiao-Juan Yu, Su-Xia Wang, Min Chen, Ming-Hui Zhao

**Affiliations:** ^1^ Renal Division, Department of Medicine, Peking University First Hospital, Beijing, China; ^2^ Institute of Nephrology, Peking University, Beijing, China; ^3^ Key Laboratory of Renal Disease, Ministry of Health of China, Beijing, China; ^4^ Key Laboratory of Chronic Kidney Disease Prevention and Treatment (Peking University), Ministry of Education, Beijing, China; ^5^ Research Units of Diagnosis and Treatment of Immune-Mediated Kidney Diseases, Chinese Academy of Medical Sciences, Beijing, China

**Keywords:** diabetic nephropathy, proteinuria, histopathology, outcome, nonproteinuric diabetic nephropathy

## Abstract

**Introduction:**

As the most common complication of diabetes mellitus (DM), diabetic nephropathy (DN) was initially considered to begin with proteinuria preceding the progression of renal insufficiency. This clinical paradigm has been questioned in the late decades, as many DM patients without proteinuria have progressive renal insufficiency. However, the characteristics of nonproteinuric DN were not fully clear yet.

**Patients and Methods:**

A total of 390 patients with renal biopsy-proven DN in our center were retrospectively recruited in the current study. Clinical and histopathological data of the patients were analyzed. We used propensity score-matching methods to address the imbalance of age, sex, and diabetes duration for comparative analyses.

**Results:**

Among all the renal biopsy-proven DN patients with renal biopsy proven DN, 18 patients were classified as nonproteinuric DN. Compared with 36 propensity score-matched proteinuric DN patients, diabetic retinopathy (DR) was less frequent in nonproteinuric DN patients (38.9% *vs.* 66.4%, p<0.05). During the follow-up of 24.0 (12.0–42.0) months, the probability of developing the end-stage renal disease (ESRD) was significantly lower in nonproteinuric DN patients than in proteinuric ones in both the propensity score-matched cohort and overall cohort (log-rank test, p<0.001 and p<0.001, respectively).

**Conclusions:**

Compared with proteinuric DN patients, DR was less frequent in nonproteinuric DN patients. Nonproteinuric DN patients had better renal outcomes than proteinuric DN patients.

## Introduction

Diabetic nephropathy (DN) is the most common complication of diabetes mellitus (DM) and the leading cause of end-stage renal disease (ESRD) in China (1–3). DN was initially considered to begin with proteinuria preceding the progression of renal insufficiency [estimated glomerular filtration rate (eGFR) <60 mL/min/1.73 m^2^]. The natural history was divided into normoalbuminuria (urinary albumin-to-creatinine ratio [UACR] <30 mg/g), microalbuminuria (UACR 30–300 mg/g), and macroalbuminuria (UACR >300 mg/g), which was mainly based on the typical progression course of type 1 DM (4).

However, this concept of the clinical paradigm has changed over the last decades, and it has been noted that DM patients without proteinuria could also have progressive renal insufficiency. Therefore, the latest diagnostic criteria for diabetic kidney disease (DKD) include low eGFR or the persistent presence of elevated urinary albumin excretion (albuminuria) (5). Nonproteinuric DKD was defined as an eGFR <60 mL/min/1.73 m^2^ with a UACR <300 mg/g (6–10). As a diagnosis term, DKD covered both clinical diagnosis and histological diagnosis (DN).

The characteristics of nonproteinuric DN patients are not yet thoroughly investigated. Previous studies showed that the renal histopathological findings of DN are heterogeneous regardless of the level of GFR or UACR (10, 11). According to the previous results, we speculated that nonproteinuric DN patients might have typical histopathological features of DN and a lower risk of CKD progression. Therefore, in the current study, using the cohort of our center and propensity score-matching methods, we investigated clinicopathological characteristics and outcomes in patients with the nonproteinuric phenotype of DN in comparison with patients with the classical proteinuric DN.

## Patients And Methods

### Patients

A total of 390 DM patients with renal biopsy-proven DN who were diagnosed from January 1, 2015, to December 31, 2020, were analyzed retrospectively. DM was defined according to the criteria proposed by the American Diabetes Association in 2017 (12). The investigation was conducted according to the Declaration of Helsinki and was approved by the Ethics Committee of Peking University First Hospital (2017-1280). Written informed consent was obtained from each participant.

**Figure 1:**
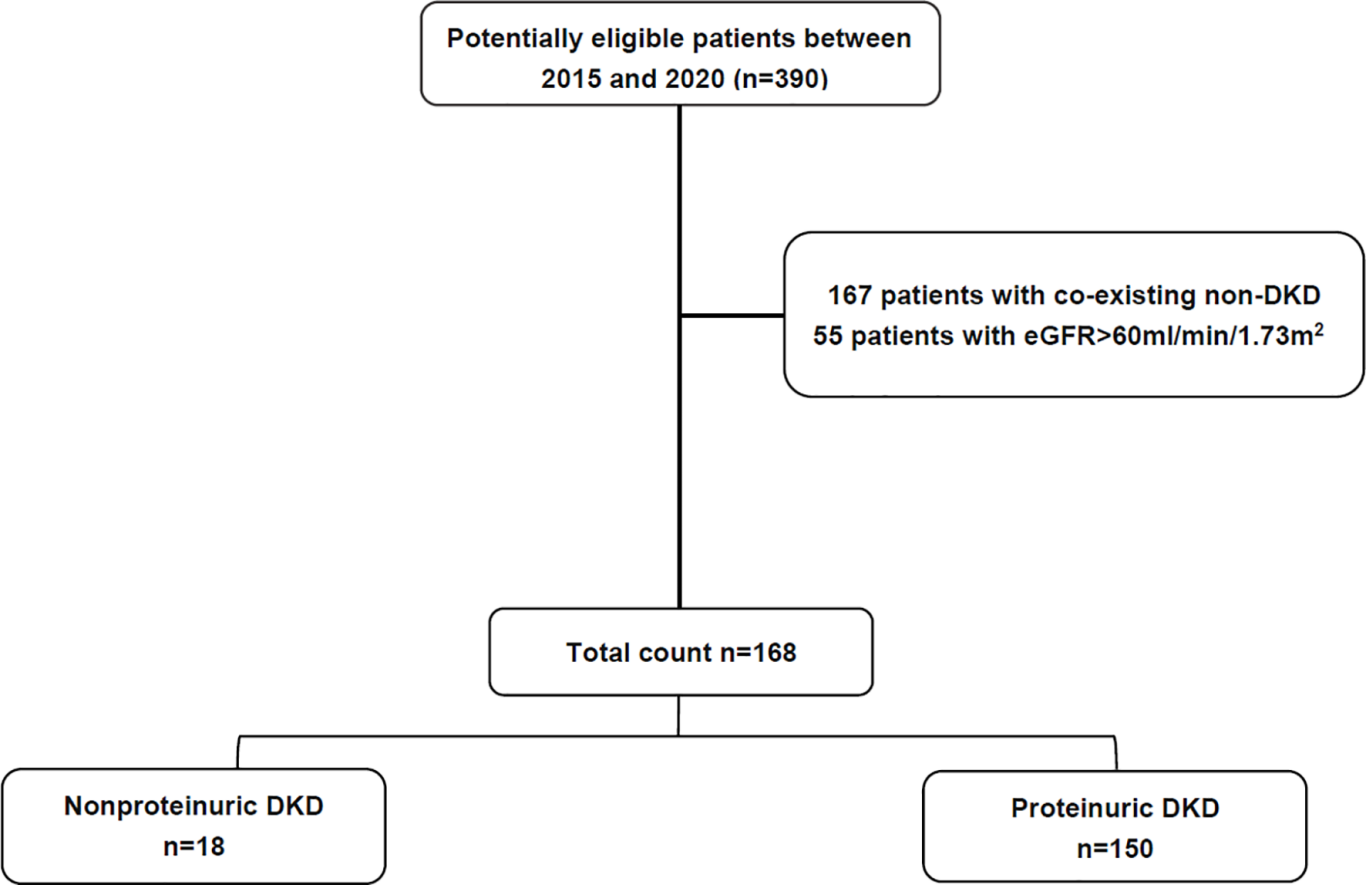
Flowchart for recruitment.

Among the 390 patients with renal biopsy-proven DN, 298 were male and 92 were female, with an age of 53.11 ± 12.59 years at renal biopsy. The median level of UACR was 2718.56 (1195.57–4897.83) mg/g (**Table 1**). Of the 390 patients, 167 patients who had coexisting non-diabetes-related renal disease, including 54 patients with membranous nephropathy, 45 patients with IgA nephropathy, 15 patients with immune complex-mediated glomerulonephritis, 10 patients with ANCA-associated glomerulonephritis, 7 patients with C3 glomerulonephritis, 6 patients with IgG4-related kidney disease and 30 patients with other renal diseases, were excluded. The comparison between patients with and without coexisting non-diabetes-related renal disease was provided in **Supplementary Table 1**, **Supplementary Figure 1**. 55/390 patients with eGFR>60 ml/min/1.73m^2^ were excluded. Ultimately, 168 patients were eligible for further analysis for different proteinuria groups. Among them, 18/168 patients were classified as nonproteinuric DN (UACR <300 mg/g) and 150/168 patients were classified as proteinuric DN (**Figure 1**).

**Table 1:** Clinical characteristics at the time of renal biopsy (n=390).

Age (years)	53.11 ± 12.59
Male	298 (76.4)
UACR (mg/L)	2718.56 (1195.57-4897.83)
Serum creatinine (μmol/L)	155.55 (104.30-272.72)
eGFR (mL/min/1.73 m^2^)	40.29 (20.24-64.24)
≥90 60-89 45-59 30-44 15-29 <15	43 (11.0) 71 (18.2) 58 (14.9) 69 (17.7) 87 (22.3) 62 (15.9)
Diabetes duration (months)	120.0 (60.0-192.0)
Diabetic retinopathy (%)	226 (57.9)
HbA1c (%)	6.7 (6.0-7.8)
Hypertension duration (months)	24.0 (1.0-114.0)

### Clinical Characteristics

The clinical data of these patients at the time of renal biopsy and during follow-up were systematically recorded, including age, sex, diabetic retinopathy (DR), use of renin-angiotensin-aldosterone system (RAAS) inhibitors, hemoglobin, serum creatinine (Scr), eGFR, serum albumin, fasting blood glucose (FBG), HbA1c, triglyceride (TG), high-density lipoprotein (HDL), low-density lipoprotein (LDL), and plasma complements. Proteinuria was expressed as the UACR. Nonproteinuric DN was defined as an eGFR <60 mL/min/1.73 m^2^ with a UACR <300 mg/g at the time of renal biopsy according to the previously described criteria (6–10). Cardiovascular disease (CVD) history was self-reported and included a history of congestive heart failure, coronary heart disease, heart attack, angina, stroke, or periphery atherosclerosis. The eGFR was calculated using the CKD-EPI equation (13). HbA1c levels were measured using a high-performance liquid chromatographic assay.

### Renal Histopathology

Renal specimens were evaluated using direct immunofluorescence (for immunoglobulins and complement components), light microscopy, and electron microscopy. Periodic acid-Schiff (PAS), silver methenamine, hematoxylin and eosin (HE), and Masson’s trichrome staining were used for light microscopy. Biopsies were scored independently by two pathologists. A standard classification system was used based on histological scores for glomerular lesions, tubulointerstitial lesions, vascular lesions and non-diabetic renal lesions (14).

Diabetic glomerulopathy is classified as class I through IV according to the Renal Pathology Society in 2010 (14). Interstitial fibrosis and tubular atrophy (IFTA) were scored semi-quantitatively based on the proportion of the tubulointerstitial compartment affected (0, none; 1, <25%; 2, 25–50%; 3, >50%). Interstitial inflammation was scored semi-quantitatively (0, absent; 1, infiltration only in areas related to IFTA; 2, infiltration in areas without IFTA). Vascular lesions were scored according to the presence of arteriolar hyalinosis and large-vessel arteriosclerosis (grades 0–1) (14). For direct immunofluorescence, the intensities of staining of immunoglobulins, complements, fibrin-associated antigen (FRA), and albumin (Alb) were semi-quantitatively graded on a scale of 0–4+.

### Outcomes

ESRD was defined as the initiation of hemodialysis/peritoneal dialysis, renal transplantation, or death due to uremia. The patients were followed up until the end of 2020 or ESRD, whichever came first. New-onset CVD events included congestive heart failure, coronary heart disease, heart attack, angina, stroke, or periphery atherosclerosis until 2020.

### Statistical Analysis

Normally distributed data were presented as mean ± standard deviation, while non-normally distributed data were presented as median values with an inter-quartile range (IQR). Categorical variables were expressed as percentages or ratios. Chi-square, one-way analysis of variance (ANOVA), and t-tests were performed as appropriate. Differences in semi-quantitative and quantitative parameters that were not normally distributed were assessed using Kruskal-Wallis or Mann-Whitney U-tests, as appropriate. Differences were considered significant if the p-value was <0.05. In the current study, the sample size of patients with nonproteinuric DN (n=18) was relatively small compared with the proteinuric DN patients (n=150). We conducted propensity score matching analysis to address the imbalance of background factors such as age, sex, and diabetes duration that affect outcomes. We matched the nonproteinuric DN group with the proteinuric DN group using propensity scores with a one-to-two nearest-neighbor caliper width of 0.01, which is the maximum allowable difference in propensity scores. Analyses were performed using the SPSS statistical software package (version 11.0; Chicago, IL, USA) and R studio 4.0.2.

## Results

### General Data of the Patients at Renal Biopsy

General data at the renal biopsy of the whole cohort of 390 DN patients were listed in **Table 1**. Among the 18 nonproteinuric DN patients, 13 were male and 5 were female, with 61.39 ± 6.11 years at the time of renal biopsy. The median duration of diabetes was 120.0 (60.0–168.0) months. Seven out of 18 (38.9%) nonproteinuric DN patients complicated with DR. Nine out of 18 (50.0%) patients had hypertension, and the median duration of hypertension was 24.0 (2.0–120.0) months. The median UACR was 147.69 (70.37–279.41) mg/g. The median Scr and eGFR levels were 201.25 (172.00–266.70) μmol/L and 28.81 (21.28–37.46) mL/min/1.73m^2^, respectively (**Table 2**).

**Table 2:** Clinical features of patients stratified by proteinuria.

	Overall cohort			Propensity score-matched cohort		
	Nonproteinuria DN	Proteinuria DN	P value	Nonproteinuria DN	Proteinuria DN	P value
	n=18	n=150		n=18	n=36	
**Age**	61.39 ± 6.11	49.80 ± 6.42	<0.001	61.39 ± 6.11	59.86 ± 7.19	0.536
**Male/Female**	13/5	113/37	0.083	13/5	24/12	0.679
**Diabetes duration (months)**	120.0 (60.0,168.0)	120.0 (72.0,192.0)	0.621	120.0 (60.0,168.0)	120.0 (84.0,216.0)	0.592
**Diabetic retinopathy (%)**	38.9	78.7	<0.001	38.9	66.4	0.031
**CVD history (%)**	44.4	44.7	1	44.4	63.9	0.173
**Hypertension duration (months)**	24.0 (2.0,120.0)	24.0 (4.0,84.0)	1	24.0 (2.0,120.0)	66.0 (24.0,240.0)	0.119
**Fasting blood glucose (mmol/L)**	5.84 (5.12,8.90)	6.38 (5.41, 7.80)	0.894	5.84 (5.12,8.90)	6.01 (5.41,7.08)	0.808
**HbA1c (%)**	6.45 (6.15,7.55)	6.60 (5.90,7.60)	0.712	6.45 (6.15,7.55)	6.40 (6.10,7.70)	0.977
**Urine NAG (U/L)**	11.20 (9.00, 14.50)	24.00 (13.25,47.00)	0.001	11.20 (9.00, 14.50)	23.80 (13.70,54.00)	0.002
**Urine α1-microglobulin (mg/L)**	51.40 (27.2,79.70)	68.10 (39.75,109.00)	0.302	51.40 (27.2,79.70)	73.65 (46.20,127.50)	0.181
**Hemoglobin (g/L)**	109.78 ± 20.52	104.69 ± 19.24	0.467	109.78 ± 20.52	105.50 ± 18.89	0.449
**Scr (μmol/L)**	201.25 (172.00,266.70)	227.92 (153.01,351.50)	0.538	201.25 (172.00,266.70)	228.30 (169.93, 349.28)	0.419
**eGFR (mL/min/1.73 m^2^)**	28.81 (21.28,37.46)	25.97 (15.28,41.60)	0.922	28.81 (21.28,37.46)	25.85 (13.37,33.08)	0.497
**Serum albumin (g/L)**	41.11 ± 3.61	31.70 ± 5.49	<0.001	41.11 ± 3.61	32.65 ± 5.81	<0.001
**Platelet (×10^9^/L)**	209.65 ± 73.64	224.39 ± 76.95	0.088	209.65 ± 73.64	190.90 ± 75.15	0.41
**Uric acid (μmol/L)**	365.22 ± 106.47	427.63 ± 116.81	0.032	365.22 ± 106.47	429.11 ± 146.73	0.107
**LDL-cholesterol (mmol/L)**	2.07 (1.71,2.37)	2.85 (2.07,3.56)	0.001	2.07 (1.71,2.37)	2.80 (2.10,3.42)	0.008
**HDL-cholesterol (mmol/L)**	0.81 (0.64,0.99)	0.93 (0.80,1.14)	0.011	0.81 (0.64,0.99)	0.92 (0.84,1.12)	0.026
**Triglyceride (mmol/L)**	2.00 (1.51, 2.92)	1.89 (1.28,2.90)	0.61	2.00 (1.51, 2.92)	1.83 (1.28,2.52)	0.428
**Serum C3**	0.94 (0.78,1.12)	0.87 (0.75,0.99)	0.303	0.94 (0.78,1.12)	0.86 (0.74,1.04)	0.266
**Serum C4**	0.27 (0.20,0.33)	0.27 (0.22,0.33)	0.687	0.27 (0.20,0.33)	0.23 (0.19,0.32)	0.443
**RAAS inhibitor**	4 (22.2%)	42 (28.0%)	0.76	4 (22.2%)	11 (30.6%)	0.519

Chi-square tests were performed in percentages or ratios variables. T-tests were performed in normally distributed variables. semi-quantitative and quantitative parameters that were not normally distributed were assessed using Kruskal-Wallis or Mann-Whitney U-tests.

Values are expressed as a mean ± standard deviation, percentage or median with upper and lower quartile or percentage.

### Comparison of Clinical Manifestations

Clinical features of patients stratified by proteinuria before and after propensity score matching are shown in **Table 2**. Compared with the 36 propensity score-matched proteinuric DN patients, DR was significantly less frequent in nonproteinuric DN patients (38.9% *vs.* 66.4%, p<0.05, respectively). Nonproteinuric DN patients showed a significantly lower level of urinary NAG and a higher level of serum albumin compared with proteinuric DN patients (11.20 [9.00–14.50] U/L *vs.* 23.80 [13.70–54.00] U/L, p<0.05; 41.11 ± 3.61 g/L *vs.* 32.65 ± 5.81 g/L, p<0.001, respectively). Significantly lower LDL-cholesterol and HDL-cholesterol levels were observed in nonproteinuric DN patients compared with proteinuric DN patients [2.07 (1.71–2.37) mmol/L *vs.* 2.80 (2.10–3.42) mmol/L, p<0.05; 0.81 (0.64–0.99) mmol/L *vs.* 0.92 (0.84–1.12) mmol/L, p<0.05, respectively]. There was no significant difference in RAAS inhibitor use between the two groups.

### Comparison of Renal Histopathological Features

Detailed renal histopathological manifestations are shown in **Table 3**. According to the international consensus classification of DN proposed in 2010, most nonproteinuric DN patients showed typical diabetic glomerulopathy, including mesangial expansion or nodular sclerosis (Kimmelstiel-Wilson lesions), 3 (16.7%), 11 (61.1%), 3 (16.7%), and 1 (5.5%) of whom were categorized as class I, class II, class III, and class IV, respectively. Varying degrees of tubulointerstitial damage were found in nonproteinuric DN patients.

**Table 3:** Renal histopathological features of patients stratified by proteinuria.

	Overall cohort			Propensity score-matched cohort			
	Nonproteinuric DN n=18	Proteinuria DN n=150	P value	Nonproteinuric DN n=18	Proteinuria DN n=36	P value
**Glomerular classification** Class I/Class II/Class III/Class IV	3/11/3/1	1/25/99/25	<0.001	3/11/3/1	0/9/19/8	0.001
**Interstitial lesions**						
**IFTA** 0/1/2/3	0/4/11/3	0/17/68/65	0.074	0/4/11/3	0/9/12/15	0.107
**Interstitial inflammation** 0/1/2	0/5/13	0/36/114	0.143	0/5/13	0/15/21	0.319
**Vascular lesions**						
**Arteriolar hyalinosis** 0 1	6 12	19 131	0.034	6 12	4 32	0.048
**Arteriosclerosis** 0 1	0 18	0 150	NA	0 18	0 36	NA
**IgG deposition (0/≥1)**	12/6	28/122	0.143	12/6	10/26	0.673
**IgM deposition (0/≥1)**	16/2	47/103	<0.001	16/2	8/28	<0.001
**IgA deposition (0/≥1)**	14/4	41/109	0.644	14/4	9/27	0.822
**C3 deposition (0/≥1)**	10/8	42/108	0.017	10/8	11/25	0.076
**C1q deposition (0/≥1)** **Alb deposition (0/≥1)**	18/0	45/105	0.007	18/0	15/21	0.001
	13/5	28/122	0.358	13/5	9/27	0.826

Values are expressed as a mean ± standard deviation, percentage or median with upper and lower quartile or percentage.

Chi-square tests were performed in percentages or ratios variables.

Compared with proteinuric DN patients, nonproteinuric DN patients had milder glomerular injuries (**Table 3**). For example, advanced DN pathology manifestations (class III and class IV) were observed in only 4/18(22.2%) of nonproteinuric DN patients, whereas they were found in 27/36(75.0%) of matched proteinuric ones. No significant difference in tubulointerstitial damage was found between the two matched groups. The proportion of patients with arteriolar hyalinosis was significantly lower in the nonproteinuric DN group than in matched proteinuric group (66.7% *vs.* 88.9%, p<0.05). All nonproteinuric and proteinuric DN patients showed arteriosclerosis in the kidneys (**Table 3**).

Regarding direct immunofluorescence, there were significantly lower proportions of IgM and C1q depositions in nonproteinuric DN patients than in matched proteinuric ones (11.1% *vs.* 77.8%, p<0.001 and 0.0% *vs.* 58.3%, p<0.05, respectively) (**Table 3**). A significantly higher proportion of C3 deposition was found in patients with proteinuria in the overall cohort (44.4% *vs.* 72.0%, p<0.05) (**Table 3**).

### Outcomes

**Figure 2:**
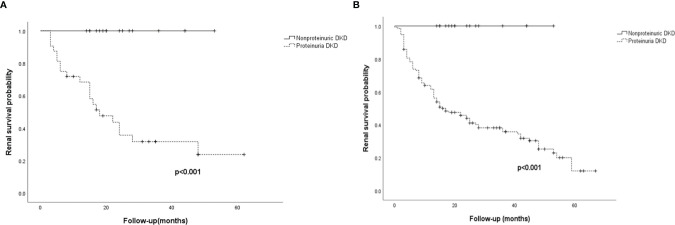
Renal survival for the 54 patients in the propensity score-matched cohort and the 168 patients in the overall cohort. **(A)** Kaplan-Meier curves of renal survival in the propensity score-matched cohort. **(B)** Kaplan-Meier curves of renal survival in the overall cohort. ESRD was defined as initiation of hemodialysis/peritoneal dialysis, renal transplantation, or death as a result of uremia. Nonproteinuric DN was defined as patients with an eGFR<60 mL/min/1.73 m^2^ without proteinuria (UACR<300 mg/g); proteinuria DN was defined as patients with an eGFR<60 mL/min/1.73 m^2^ and proteinuria (UACR>300 mg/g).

During a median follow-up duration of 24.0 (12.0–42.0) months, none of the nonproteinuric DN patients progressed to ESRD, whereas 21/36 (65.6%) of the matched proteinuric DN patients progressed to ESRD. Among the patients with proteinuria from the overall cohort, 92/150 (61.3%) progressed to ESRD. Kaplan-Meier analysis showed that the probability of developing ESRD was significantly lower in nonproteinuric DN patients than in proteinuric ones in both the propensity score-matched cohort and overall cohort (log-rank test, p<0.001 and p<0.001, respectively) (**Figure 2**). Only 1/18 patients with nonproteinuric DN and 22/150 patients with proteinuria DN had new-onset CVD in the current study (P>0.05), which might be due to the relatively short follow-up.

## Discussion

DN is the leading cause of ESRD and is associated with increased cardiovascular morbidity and all-cause mortality (15–17). Traditionally, persistent microalbuminuria has been considered the first clinical sign of DN, inevitably progressing to macroalbuminuria and subsequent renal dysfunction (18). However, over recent decades, there has been increasing recognition that GFR reduction may precede the development of proteinuria in several patients with diabetes (6–8, 19, 20). These patients were therefore defined as nonproteinuric DKD/DN. The prevalence of proteinuric DKD declined, while the prevalence of nonproteinuric DKD increased, attributable to a higher rate of RAAS inhibitors prescription (21). Although the paradigm has been renewed, the characteristics of nonproteinuric DN have not been thoroughly investigated.

In patients with DKD, the prevalence of nonproteinuria varies between 20% and 40% (22, 23). In the current study, a total of 18/223 (8.1%) DN patients were classified as nonproteinuric DN, which was lower than that in previous reports. Of the patients with reduced eGFR (<60 mL/min/1.73 m²) from the National Health and Nutrition Examination Survey (NAHNES III) in 2003, 81% had nonproteinuric DKD, and only 19% had proteinuria (19). In the UK Prospective Diabetes Study (UKPDS-74), during 15 years of follow-up in 4,006 patients with type 2 diabetes, 1,132 (28.3%) developed renal impairment. Of the latter, 575 (50.8%) patients were classified as nonproteinuric DKD (24). We have noted that all patients in the current study underwent renal biopsy, which was not highly recommended in nonproteinuric DKD patients unless they were suspected of having either superimposed non-diabetic kidney disease or *de novo* non-diabetic kidney disease (25). The relatively lower prevalence of nonproteinuric DN patients in the current study might be associated with the lower rate of renal biopsy in this subgroup of patients. In summary, the prevalence of nonproteinuric DKD is not low. The traditional nonproteinuric DKD should also be paid attention and concern on, mainly due to lower eGFR and renal insufficiency.

Compared with proteinuric DN patients, a significantly lower proportion of DR in nonproteinuric DN patients was found in both the overall and the matched cohorts. The prevalence of DR in patients with nonproteinuric DKD varies across studies. A study from RIACE with 2,959 DKD patients found that 2,028 (68.5%) patients did not have DR, and 538 patients (18.2%) showed both proteinuria and retinopathy (26). The varying prevalence of DR suggests that the development of nonproteinuric DKD may be independent of diabetic microangiopathic lesions (19, 23).

Only a limited number of studies have investigated the renal histopathological features of nonproteinuric DN. Results from previous biopsy-based studies were inconsistent, which may be due to the small sample size and the timing of renal biopsy. Studies of the renal histopathology in patients with type 2 DM showed that nonproteinuric patients had less frequent typical glomerular injuries. The findings were not consistent for tubulointerstitial and arterial injuries (11, 27, 28). Yamanouchi et al. reported that patients with nonproteinuric DN have both milder glomerular injuries and tubulointerstitial injuries (10). In the current study, consistent with previous reports, most of the nonproteinuric DN patients showed typical but milder glomerular injuries, including mesangial expansion and nodular sclerosis (Kimmelstiel-Wilson lesions), while tubulointerstitial injuries were heterogeneous. More importantly, these results suggest that typical glomerular injuries may precede overt proteinuria in DN. For immunofluorescence, there was a significantly lower proportion of IgM and C1q deposition in nonproteinuric DN patients compared with matched proteinuric DN patients. A higher proportion of C3 deposition was found in patients with proteinuria in the overall cohort. Previous studies have shown that complement deposition in renal histopathology is associated with severe kidney damage in DN patients (29, 30). Persistent proteinuria may induce local complement activation and aggravate renal injury. The pathogenic role of complement overactivation warrants further investigation.

In the current study, the renal outcome was more favorable in nonproteinuric DN patients than those with proteinuria. None of the nonproteinuric DN patients progressed to ESRD. These results were consistent with previous studies (31, 32). Proteinuria remains a crucial independent predictor of eGFR decline in DM patients, especially those with low eGFR. However, even if the risk for ESRD was low, nonproteinuric patients showed an equal or even higher risk of CVD morbidity and mortality than those with proteinuria (33–37). The results suggest that nonproteinuric DN may represent a distinct phenotype, with macroangiopathic and tubulointerstitial lesions instead of microangiopathic lesions involved in the underlying pathology. Close attention and care for CVD morbidity and mortality in these patients are needed.

This study has some limitations. First, the sample size was small, and the follow-up duration was short for assessing the probability of developing ESRD. The current study was a single-center study that recruited only 18 nonproteinuric DN patients. Therefore, the true prevalence of nonproteinuric DKD cannot be accurately assessed. Second, there was an inevitable bias in patients receiving renal biopsy. Third, we only referred to Chinese DN patients in the current study. Studies involving multi-ethnic and multi-center are needed.

## Conclusion

In conclusion, compared with proteinuric DN patients, DR was less frequent in nonproteinuric DN patients. Nonproteinuric DN patients had better renal outcomes than proteinuric patients. Multicenter studies with larger sample sizes are needed to further understand nonproteinuric DN.

## Data Availability Statement

The raw data supporting the conclusions of this article will be made available by the authors, without undue reservation.

## Ethics Statement

The studies involving human participants were reviewed and approved by Ethics Committee of Peking University First Hospital (2017-1280). The patients/participants provided their written informed consent to participate in this study.

## Author Contributions

D-YC and MC designed the study. D-YC, M-RL, X-JY, and S-XW contributed data. D-YC and MC drafted the analysis plan. D-YC performed the statistical analysis. D-YC wrote the manuscript. MC and M-HZ revised the manuscript and supervised the study. MC is the guarantors of this work and, as such, had full access to all the data in the study and take responsibility for the integrity of the data and the accuracy of the data analysis. All authors contributed to the article and approved the submitted version.

## Funding

This study is supported by a grant from China International Medical Foundation-Renal Anemia Fund, a grant from National Key Research and Development Program (No. 2016YFC1305405), the grants from the National Natural Science Found (No. 82070748, 82090020 and 82090021) and CAMS Innovation Fund for Medical Sciences (2019-I2M-5-046).

## Conflict of Interest

The authors declare that the research was conducted in the absence of any commercial or financial relationships that could be construed as a potential conflict of interest.

## Publisher’s Note

All claims expressed in this article are solely those of the authors and do not necessarily represent those of their affiliated organizations, or those of the publisher, the editors and the reviewers. Any product that may be evaluated in this article, or claim that may be made by its manufacturer, is not guaranteed or endorsed by the publisher.

## Supplementary Material

Supplementary Table 1 Clinical features of DN patients with non-diabetic renal disease (NDRD) and without NDRD. Values are expressed as a mean ± standard deviation, percentage or median with upper and lower quartile or percentage. Chi-square tests were performed in percentages or ratios variables. T-tests were performed in normally distributed variables. semi-quantitative and quantitative parameters that were not normally distributed were assessed using Kruskal-Wallis or Mann-Whitney U-tests.
