# Supplementary material for: Clinical and Pathological Characteristics of Patients With Nonproteinuric Diabetic Nephropathy
Source: Front Endocrinol (Lausanne). 2021 Oct 26;12:761386. doi: 10.3389/fendo.2021.761386 (PMC8576342; doi:10.3389/fendo.2021.761386)
Supplement: Supplementary Table 1 — Clinical features of DN patients with non-diabetic renal disease (NDRD) and without NDRD. Values are expressed as a mean ± standard deviation, percentage or median with upper and lower quartile or percentage. Chi-square tests were performed in percentages or ratios variables. T-tests were performed in normally distributed variables. semi-quantitative and quantitative parameters that were not normally distributed were assessed using Kruskal-Wallis or Mann-Whitney U-tests. [file Table_1.doc]

Supplementary Table 1. Clinical features of DN patients with non-diabetic renal disease (NDRD) and without NDRD

|  | **DN with NDRD**  **n=167** | **DN without NDRD**  **n=223** | **P value** |
| --- | --- | --- | --- |
| **Age** | 56.75±12.26 | 50.39±12.16 | <0.001 |
| **Male/Female** | 131/36 | 167/56 | 0.413 |
| **Diabetes duration (months)** | 120.0(36.0,192.0) | 120.0(72.0,180.0) | 0.333 |
| **Diabetic retinopathy (%)** | 39.5 | 68.6 | <0.001 |
| **Hypertension duration(months)** | 36.0(1.0,120.0) | 24.0(2.0,96.0) | 0.603 |
| **Cardiovascular disease (%)** | 33.5 | 42.2 | 0.562 |
| **Fasting blood glucose (mmol/L)** | 6.08(5.36,7.57) | 6.41(5.32, 8.14) | 0.645 |
| **HbA1c (%)** | 6.70 (6.15,7.55) | 6.70(5.95,8.00) | 0.452 |
| **Urine NAG (U/L)** | 23.00(12.90, 46.00) | 22.90(12.50,41.00) | 0.558 |
| **Urine α1-microglobulin (mg/L)** | 48.40(20.7,91.00) | 45.35(22.20,91.00) | 0.726 |
| **Hemoglobin (g/L)** | 119.98±23.63 | 111.15±22.83 | 0.001 |
| **Scr (μmol/L)** | 139.10(92.80,2639.50) | 175.80(108.4,295.00) | 0.017 |
| **eGFR (mL/min/1.73 m^2^)** | 46.50(21.52,68.67) | 36.89(19.82,60.98) | 0.039 |
| **Serum albumin (g/L)** | 30.42±3.38 | 33.21±6.12 | <0.001 |
| **Platelet (×10^9^/L)** | 234.98±89.31 | 220.94±73.91 | 0.012 |
| **Uric acid(μmol/L)** | 411.36±119.40 | 407.94±112.26 | 0.788 |
| **LDL-cholesterol(mmol/L)** | 2.94(2.14,3.92) | 2.67(2.04,3.47) | 0.097 |
| **HDL-cholesterol (mmol/L)** | 0.97(0.84,1.18) | 0.94(0.81,1.13) | 0.212 |
| **Triglyceride (mmol/L)** | 1.89(1.39, 2.66) | 1.79(1.25,2.75) | 0.286 |

Chi-square tests were performed in percentages or ratios variables. T-tests were performed in normally distributed variables. semi-quantitative and quantitative parameters that were not normally distributed were assessed using Kruskal-Wallis or Mann-Whitney U-tests.

Values are expressed as a mean±standard deviation, percentage or median with upper and lower quartile or percentage.
