# Supplementary figures and images for: Clinical and Pathological Characteristics of Patients With Nonproteinuric Diabetic Nephropathy
Source: Front Endocrinol (Lausanne). 2021 Oct 26;12:761386. doi: 10.3389/fendo.2021.761386 (PMC8576342; doi:10.3389/fendo.2021.761386)

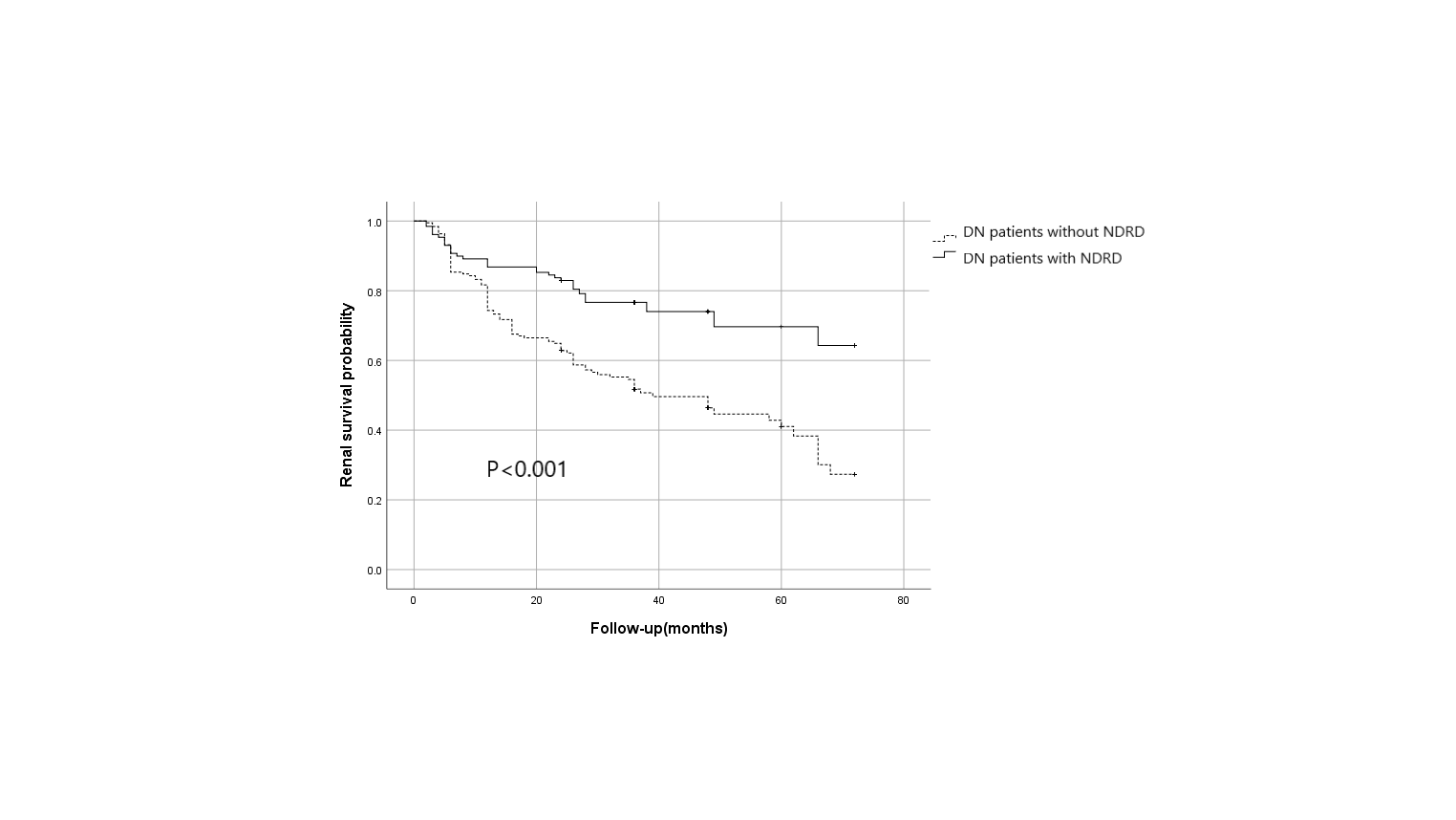

Supplement: Supplementary file 2 [file Image_1.tiff]
